# Probiotic Yeast *Saccharomyces*: Back to Nature to Improve Human Health

**DOI:** 10.3390/jof8050444

**Published:** 2022-04-24

**Authors:** Rameesha Abid, Hassan Waseem, Jafar Ali, Shakira Ghazanfar, Ghulam Muhammad Ali, Abdelbaset Mohamed Elasbali, Salem Hussain Alharethi

**Affiliations:** 1 Department of Biotechnology, University of Sialkot, Sialkot 51310, Pakistan; jafaraliqau@gmail.com; 2 National Agriculture Research Center, National Institute of Genomics and Agriculture Biotechnology (NIGAB), Islamabad 44100, Pakistan; shakira_akmal@yahoo.com; 3 Department of Biological Sciences, Muslim Youth University, Islamabad 44100, Pakistan; pro.vc@myu.edu.pk; 4 Pakistan Agricultural Research Council (PARC) 20, Ataturk Avenue, G-5/1, Islamabad 44000, Pakistan; drgmali5@gmail.com; 5 Department of Clinical Laboratory Science, College of Applied Sciences-Qurayyat, Jouf University, Al-Jouf P.O. Box 2014, Saudi Arabia; 6 Department of Biological Science, College of Arts and Science, Najran University, Najran 66262, Saudi Arabia; shalharthi@nu.edu.sa

**Keywords:** *S. cerevisiae* var. *boulardii*, gastrointestinal diseases, microbiota, intrinsic and extrinsic factors, probiotic yeast

## Abstract

*Saccharomyces cerevisiae* var. *boulardii* is best known for its treatment efficacy against different gastrointestinal diseases. This probiotic yeast can significantly protect the normal microbiota of the human gut and inhibit the pathogenicity of different diarrheal infections. Several clinical investigations have declared *S. cerevisiae* var. *boulardii* a biotherapeutic agent due to its antibacterial, antiviral, anti-carcinogenic, antioxidant, anti-inflammatory and immune-modulatory properties. Oral or intramuscular administration of *S. cerevisiae* var. *boulardii* can remarkably induce health-promoting effects in the host body. Different intrinsic and extrinsic factors are responsible for its efficacy against acute and chronic gut-associated diseases. This review will discuss the clinical and beneficial effects of *S. cerevisiae* var. *boulardii* in the treatment and prevention of different metabolic diseases and highlight some of its health-promising properties. This review article will provide fundamental insights for new avenues in the fields of biotherapeutics, antimicrobial resistance and one health.

## 1. Introduction

According to the latest definition of the World Health Organization, probiotics are active microbes that stimulate the growth of other probiotic bacteria in the gut and possess beneficial health effects to the host [1]. These microorganisms are able to produce anti-carcinogenic, antioxidant and anti-mutagenic agents and induce protection against different bacterial diseases including diarrhea and respiratory tract infections. *Saccharomyces cerevisiae* var. *boulardii* is the most significant probiotic yeast species. *S. cerevisiae* var. *boulardii* is a eukaryotic organism that has been used in scientific investigations since the time of its discovery [2]. This model organism has unique importance because of its alterable and flexible genome. The genome of *S. cerevisiae* var. *boulardii* was completely sequenced in 1950 and a genome size of approximately 11.3 Mb was reported. It has approximately 6000 genes and 275 additional tRNA genes. Almost 23% of the *S. cerevisiae* var. *boulardii’s* genome is homologous to the hominid genome. This specific yeast is best known for its role in treating gastrointestinal diseases [3,4]. 

*S. cerevisiae var. boulardii* has gained the importance of the scientific community due to the production of different bioactive compounds [5]. This specie is an excellent protein source with high amino acid content, which is essential for the production of various foods and cosmetic supplements [6]. *S. cerevisiae* var. *boulardii* is also responsible for the formation of glutathione, an important antioxidant used in the food and drug industry [7]. The inactivated cells of *S. cerevisiae* var. *boulardii* are used as a rich protein source in probiotic feed supplements. Despite its high protein content and antioxidant nature, the thick and indigestible cell wall and high nucleic acid content limit the use of inactivated cells of *S. cerevisiae* var. *boulardii* in human food and nutrition. It can enhance its antioxidant properties by increasing the production of phytochemical constituents, such as isoflavones. *S. cerevisiae* var. *boulardii* is used preferably due to its unique digestible properties of starch and proteins. Reduction in trypsin-inhibitor activity and phytic acid content is responsible for its digestible behavior [8]. 

The oval to round cell shape of *S. cerevisiae* var. *boulardii* is composed of approx. 3 µm thickness and 2.5–10.5 µm length. This yeast is able to reproduce sexually and asexually by budding and unification [8]. The cell wall of *S. cerevisiae* var. *boulardii* is composed of a rigid inner polysaccharide layer with a 1,3-β-glucan branched structure while the outer layer is made up of mannoproteins. The total mass of *S. cerevisiae* var. *boulardii* in terms of dry weight is almost 30% and the estimated total polysaccharide and protein contents are 85% and 15%, respectively. Biochemical characterization of *S. cerevisiae* var. *boulardii* confirmed the presence of glucose, mannose and *N*-acetylglucosamine up to 90%, 20% and 2%, respectively. Glucose to glucose interaction is associated with β-1,3 and β-1,6 linkages. β-1,3 glucan is responsible for the elasticity and strength of the yeast cell wall. The lateral cell wall of *S. cerevisiae* var. *boulardii* is composed of straight chitin chains of 1–2% of total dry weight [9]. 

The nutritional value of *S. cerevisiae* var. *boulardii* is enhanced due to the presence of different minerals, vitamins and antioxidant compounds. Dietary yeast is composed of iron, manganese and copper, some trace minerals are also reported, i.e., ferric, manganic sulfate and cupric acetate [10]. Studies suggested that several toxic metals are easily accumulated by *S. cerevisiae* var. *boulardii*_,_ which includes lead, cadmium, arsenic and mercury [11]. Nutritional yeast has the ability to enhance the energy level in an individual because of the presence of non-proteinaceous amino acids, proteinaceous amino acids and vitamin B, such as biotin, doxine, thiamin, vitamin B12 and riboflavin. It can also reduce antinutrient phytate levels and enhance the synthesis of folate. *S. cerevisiae* var. *boulardii* can also protect from bacterial infections along with increasing the glucose sensitivity to enhance the growth of skin, nails and hair [5]. 

**Figure 1:**
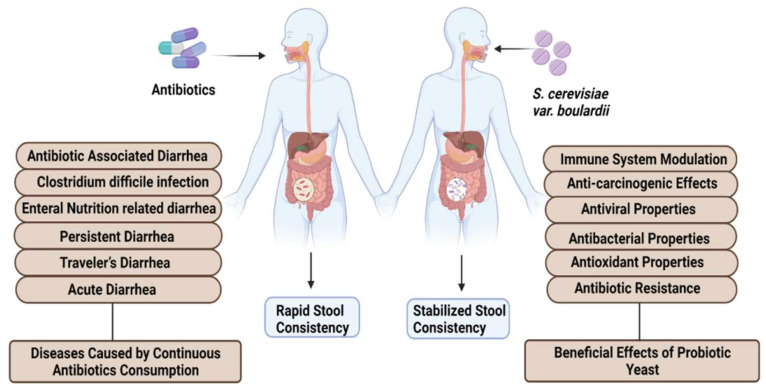
Health-promoting effects of *S. cerevisiae* var. *boulardii*.

Recently, it was observed that medical professionals are using nonpathogenic *S. cerevisiae* var. *boulardii* in the treatment of gut-related diseases. Clinical studies claimed that oral administration of *S. cerevisiae* var. *boulardii* can treat multiple gastrointestinal diseases including Traveler’s diarrhea [12], AIDS-associated diarrhea [13], antibiotic-associated diarrhea [14], *Clostridium difficile-*associated syndrome [15], Irritable Bowel Syndrome [16] and Crohn’s disease [17] (Figure 1). This yeast can be used for the treatment alone or can be administered in combination with other probiotics resulting in enhanced treatment efficiency. One hundred grams per day consumption of *S. cerevisiae* var. *boulardii* can induce beneficial effects on human health. *S. cerevisiae* var. *boulardii* cells have the ability to stick on the gastric and intestinal linings of the mucosa and actively prevail in the gastrointestinal tract of animals and humans [18]. The antineoplasmic effects of *S. cerevisiae* var. *boulardii* were reported with major findings. Oral administration of *S. cerevisiae* var. *boulardii* can inactivate epidermal growth factor receptor (EFR), which can further suppress EGFR-Erk and EGFR-Akt pathways resulting in induced apoptosis in tumor cells and reducing the level of cell colony formation and cancer cell proliferation. In vitro study claimed that *S. cerevisiae* var. *boulardii* consumption can inhibit the expression of HER2, HER-3 and IGF-1R genes which leads to the prevention of intestinal neoplasia [19]. Diarrhea caused by the continuous use of antibiotics can also be treated by *S. cerevisiae* var. *boulardii* in adults and children (Figure 1) [20]. A study claimed that this yeast can also effectively work against chronic permeability in patients with Crohn’s disease when administered orally for 3 months [21]. In HIV-linked diarrhea, exposure to a 3 g per day dose of *S. cerevisiae* var. *boulardii* can produce beneficial health effects [22]. The dose of *S. cerevisiae* var. *boulardii* in case of chronic diseases should be increased to meet the treatment criteria. Recently, an upsurge in multidrug-resistant organisms is reported due to the excessive consumption of antimicrobials [23]. Global healthcare authorities are trying to create awareness all over the globe via antibiotic stewardship programs, but the severity of antimicrobial resistance is continuously increasing [24]. To cope with this alarming situation, probiotics, especially *S. cerevisiae* and *S. cerevisiae* var. *boulardii* yeast, can be considered as an alternative method for the treatment of bacterial and fungal infections. A number of research and review articles describing the probiotic potentials of yeast and bacteria have been published in the last decade. A comprehensive review is needed to highlight the probiotic potential of *S. cerevisiae* var. *boulardii* in various aspects. Therefore, the aim of this review is to explore the diverse probiotic potential of *S. cerevisiae* var. *boulardii* through the combination of different meta-analyses. Utilization of *S. cerevisiae* var. *boulardii* as an alternate to antibiotics for the treatment and eradication of different metabolic diseases is investigated. Moreover, details of commercially available probiotic strains of *S. cerevisiae* var. *boulardii* and their clinical and beneficial detailed effects are provided. Finally, the significance of *S. cerevisiae* var. *boulardii* against cancer signaling cascades and safety attributes regarding its consumption among humans and livestock animals are thoroughly discussed. 

## 2. Enzymatic Potential of *S. cerevisiae* var. *boulardii*

**Figure 2:**
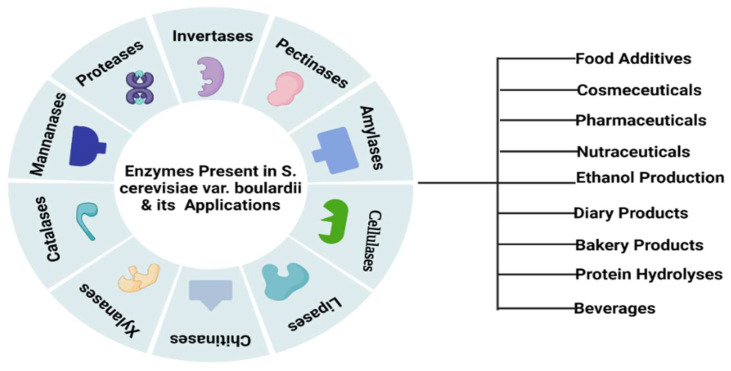
Industrial significance of *S. cerevisiae* var. *boulardii-*based enzymes.

*S. cerevisiae* var. *boulardii* can produce different enzymes which play a significant role in various industrial processes (Figure 2). Some active enzymes, i.e., maltase and invertase, have the potential to enhance the flavor of fermented products specifically in the food industry. Maltase is responsible for the conversion of malt sugar into normal sugar while invertase converts granulated sugar into regular sugar. Another enzyme, zymase, transforms normal sugar into CO_2_ and alcohol [25]. *S. cerevisiae* var. *boulardii* is able to produce intestinal enzymes including amylase, protease, cellulase and lipase and is unable to synthesize galactosidase, DNAase and gelatinase (Figure 2) [26]. *S. cerevisiae* var. *boulardii* has antibacterial properties due to the presence of extracellular protease enzymes and cell surface hydrophobicity [27]. This yeast enhances the concentration of the enzymes by the production of polyamines that trigger the cells of the intestine. Cell surface hydrophobicity is responsible for the adherence of *S. cerevisiae* var. *boulardii* yeast to the cell wall lining of the human intestine. *S. cerevisiae* var. *boulardii* is critically responsible for the production of ethanol in anaerobic conditions. This species can also show tolerance against a high level of ethanol and gastric discharge, including bile salts and intestinal acids, hence, eliminating toxic bacterial strains from the host body in the form of fecal matter [28]. Interestingly, this yeast can work against both Gram-positive and -negative bacteria and boost the host immunity. Despite its antibacterial properties, *S. cerevisiae* var. *boulardii* also showed resistance against all broad and narrow-spectrum antibiotic drugs and does not disturb the normal microbiota of the gastrointestinal tract of the host [28]. 

## 3. Factors Responsible for the Efficiency of *S. cerevisiae* var. *boulardii* as a Probiotic

Probiotics are being used to enhance treatment efficacy and to produce significant health benefits. *S. cerevisiae* var. *boulardii* is a unicellular, cost-effective active yeast species that has probiotic potential and is often used as a nutritional additive [29]. Different modes of actions were observed in favor of the host and against the antigenic microorganisms which include luminal action: (1) Antimicrobial activity: (a) Reduction in the intestinal bacterial growth [30], (b) lowering of gastrointestinal translocation of microbes [31], (c) nullifying the effect of bacterial pathogenicity [32], (d) reducing the binding affinity of the host cell with the bacterial population [33]. (2) Antitoxin effects: (a) obstructing the pathogenic receptor active sites [30], (b) enhancing the production of antibodies against *Clostridium difficile* toxin A [34], (c) mediating the synthesis of the phosphatases enzyme against *Escherichia coli (E. coli*) [35], (d) cleavage of pathogenic enzymatic proteins [35]. (3) Trophic action associated with intestinal linings: (a) reducing the expression of tumor necrosis factor-alpha (TNFα) gene and inhibiting programmed cell death [36], (b) enhancing the synthesis of glycoprotein in the intestinal brush border [37], (c) inducing the production of intestinal polyamines [37], (d) repairing fluid transport pathways [37], (e) stimulating the production of membrane enzymes (28). (4) Mediation of immune system: (a) stimulating the production of regulatory T cells [32], (b) enhancing the level of IgG antibody against *Clostridium difficile* toxin A [34], (c) improving the adherence of WBCs (White Blood Cells) to the endothelial cells [38,39]. 

Probiotics have gained global beneficial additive status to use as a potential feed supplement [40]. Human probiotic administration is based on the development and viability of probiotics in the intestinal lumen of the host organisms. Probiotic yeast has more survival chances in the stomach due to the presence of digestive enzymes, bile and gastrointestinal juices in comparison to probiotic bacteria [41]. The Food and Drug Administration (FDA) has approved certain probiotic strains which are potentially used for the benefit of humans, but *S. cerevisiae* and *S. cerevisiae* var. *boulardii* are the only probiotic yeast species that are commercially used for human benefits (Table 1) [42]. 

*S. cerevisiae* var. *boulardii* has surpassed the affectivity of the commonest probiotic bacteria, i.e., lactobacillus due to its resistance against different antibiotics [43]. *S. cerevisiae* var. *boulardii* can be administered to patients as an alternative source of antibiotics due to its outrageous antibacterial properties. Probiotic consumption can also reduce the pathogenicity of harmful microbes present in the human gut [44]. The different strains of *Saccharomyces sp.*, including *S. boulardii*, *S. cerevisiae*, and *S. unisporus*, also showed antibacterial and antiviral properties. These strains were used to enhance the probiotic potential of different human food supplements. These probiotic strains are also effective against acute and chronic diarrhea. The combination of *S. cerevisiae* var. *boulardii* with other probiotics of the same or different genus can also enhance the efficacy of human feed supplements [45]. 

**Figure 3:**
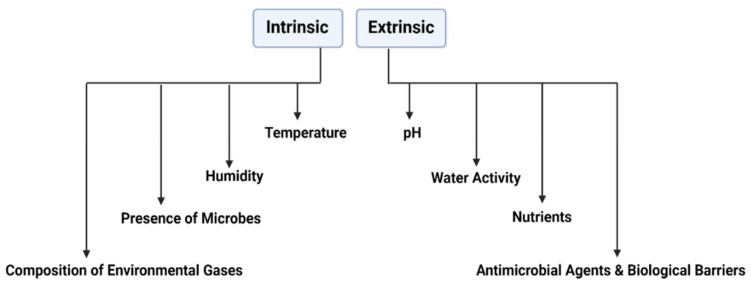
Factors affecting the efficacy of *S. cerevisiae* var *boulardii*.

Several intrinsic and extrinsic factors are directly implicated in the efficacy of *S. cerevisiae* var. *boulardii* as a probiotic (Figure 3).

### 3.1. Temperature Fluctuations

*S. cerevisiae* var. *boulardii* strains can work effectively at a temperature range of 22–30 °C (Table 2), while other *S. cerevisiae* var. *boulardii* strains are functional at 37 °C temperature and some can survive below 20 °C temperature. As a probiotic, *S. cerevisiae* var. *boulardii* is present in the form of capsules. The heat-dried *S. cerevisiae* var. *boulardii* capsules could not survive at 25 °C after opening due to their reduced potency. They retain their efficacy when stored at a 4 °C refrigerator. Lyophilized *S. cerevisiae* var. *boulardii* capsules can survive at room temperature and are viable for 1 year approximately. Studies suggested that *S. cerevisiae* var. *boulardii* can grow best at 37 °C. However, the death phase of this yeast usually appeared at 55–56 °C [46,47]. 

### 3.2. Water Activity a_w_ and Relative Humidity

Water activity a_w_ and relative humidity can produce synergistic effects. Survival of *S. cerevisiae* var. *boulardii* can be influenced by water activity. A study reported that the cells of *S. cerevisiae* var. *boulardii* showed a 0.98% value of water activity when refrigerated at −20 °C, which ultimately increased the survival rate of the yeast *S. cerevisiae* var. *boulardii* (Table 2). However, reduced water activity conditions can deteriorate the viability of *S. cerevisiae* var. *boulardii’s* cells. Water activity can also be influenced via relative humidity, specifically in the case of opened or uncovered foods. The viability of *S. cerevisiae* var. *boulardii* is reduced when the rate of water activity and environmental humidity decreases [48].

### 3.3. pH and Acidity 

*S. cerevisiae* var. *boulardii* showed resistance against less pH and more acidic conditions. However, some yeast species are too fragile to bear such a hard environment. The ideal pH range for *S. cerevisiae* var. *boulardii* development and maturation is 2–8 (Table 2). Yeast species belonging to a genus other than *Saccharomyces* showed tolerance against extreme acidic and alkaline environments. Overall functionality of the yeast is better in the lyophilized form [29,47,51].

### 3.4. Antimicrobial Agents

*S. cerevisiae* var. *boulardii* showed antimicrobial properties due to these subsequent reasons: (i) synthesis of the extracellular enzyme, i.e., protease, it aids in the formation of colonic mucosa, (ii) excretion of toxins and SO_2_ gas, it can halt the efficacy of toxins released by *Clostridium difficile*, (iii) secretion of enzyme-based proteins, (iv) cell surface hydrophobicity and autoagglutination, it is responsible for the attachment of *S. cerevisiae* var. *boulardii* to the patient’s intestinal lining. The viability of different Gram-positive and -negative bacteria can easily be reduced by the negative influence of *S. cerevisiae* var. *boulardii* on the host organism [26].

### 3.5. Nutrient Media for the Growth of S. cerevisiae var. boulardii

Yeast can grow on different nutrient media including Yeast extract peptone dextrose media, Sabouraud dextrose agar, but Oxytetracyclin yeast agar media (OGA) is considered as the best medium for its growth (Table 2). Standard OGA media can be prepared by adding 8 g of yeast extract, 9 g of glucose, 12 g of nutrient agar and 0.1 uL of oxytetracycline in 500 mL of distilled H_2_O. Carbon (glucose, maltose, sucrose and fructose), nitrogen (Urea, peptone and powdered yeast extract) and a trace amount of minerals (zinc, copper, magnesium, sulfur) are required to enhance the growth of *S. cerevisiae* var. *boulardii* [49,50]. All these factors play a significant role in maintaining the viability of *S. cerevisiae* var. *boulardii.* The potential of this beneficial yeast may be disturbed when the optimum conditions of both intrinsic and extrinsic factors changes.

## 4. Clinical Significance of *S. cerevisiae* var. *boulardii* as a Probiotic in Acute and Chronic Diseases

### 4.1. Acute Diseases 

#### 4.1.1. Antibiotic-Associated Diarrhea

Antibiotic-associated diarrhea (AAD) occurs due to the continuous consumption of antibiotics for a longer period. The use of probiotics, mainly *S. cerevisiae* var. *boulardii,* is the commonest method for the treatment against AAD. A total of 8 (80%) out of 10 (100%) controlled experiments confirmed the efficacy of *S. cerevisiae* var. *boulardii* for the prohibition of AAD specifically in adult patients (Table 3). The beneficial impact of *S. cerevisiae* var. *boulardii* and the relative decline in AAD comparable to the control are categorized in the range of 7.4% and 25%, respectively [29]. The affectivity of *S. cerevisiae* var. *boulardii* against AAD in the pediatric population has also shown positive outcomes. Results of two meta-analyses confirmed the potential of *S. cerevisiae* var. *boulardii* against AAD with a pooled risk ratio of 0.47 and 0.43 and a 95% confidence interval [52].

#### 4.1.2. Clostridium Difficile Infection (CDI)

*Clostridium difficile (C. difficile)* is a Gram-positive anaerobic rod-shaped bacteria that may cause antibiotic-associated *Clostridium difficile* diarrhea. It is responsible for the colon infection, it shows diarrhea (mild) to colon damage (severe) symptoms. Meta-analysis of six randomized control trials of different *Saccharomyces* strains including *S. cerevisiae* var. *boulardii* showed efficacy against CDI with a total risk ratio of 0.59 [53].

**Figure 4:**
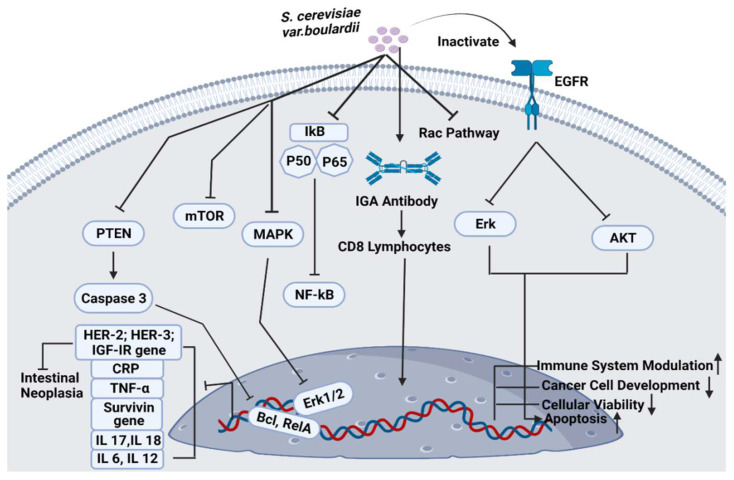
Pathways associated with *S. cerevisiae* var. *boulardii*.

The beneficial impact of *S. cerevisiae* var. *boulardii* and the relative decline in CDI comparable to the control were categorized within the range of 19% and 33.3%, respectively (Table 3). *S. cerevisiae* var. *boulardii* can prevent diarrhea caused by toxin A. It can also suppress colon inflammation and can block the intestinal toxin receptor sites via protease liberation. It can modulate the immune response by stimulating the production of IgA immunoglobulins. Moreover, probiotic use can block the activation of several kinases, Erk1/2 and interleukin 8 expression (Figure 4) [53].

#### 4.1.3. Acute Diarrhea

*S. cerevisiae* var. *boulardii* administered to patients involved in two randomized control group trials showed clinical efficacy against acute diarrhea as compared to the control (Table 3). *S. cerevisiae* var. *boulardii* consumption among 100 patients of an age less than 15 for 7 days resulted in reduced stool frequency and stabilized the normal stool condition [54]. A meta-analysis conducted among more than 600 patients that administered the *S. cerevisiae* var. *boulardii* probiotic strains for 60 days significantly reduce the rapid stool frequency [55]. Another meta-analysis of seven randomized controlled trials claimed to stabilize the childhood diarrhea consistency within 24 h as compared to the placebo treatment [56].

#### 4.1.4. Persistent Diarrhea

Two randomized controlled trials suggested that *S. cerevisiae* var. *boulardii* significantly enhances treatment efficacy specifically in children with persistent diarrhea (Table 3). The beneficial impact of *S. cerevisiae* var. *boulardii* and the relative decline in persistent diarrhea comparable to the control was 50%, respectively. However, a meta-analysis of *S. cerevisiae* var. *boulardii* against persistent diarrhea among pediatric and young populations has not been performed up till now [57]. 

#### 4.1.5. Enteral Nutrition-Related Diarrhea

**Figure 5:**
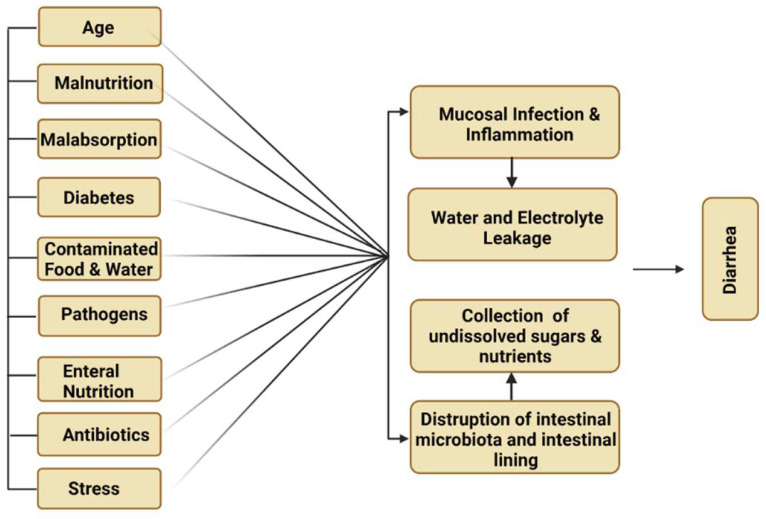
Major causes of diarrhea.

Diarrhea is the major complication associated with total enteral nutrition (TEN) and can also cause fluctuations in short-chain fatty acids (SCFA). Diabetes, gastrointestinal infection and malabsorption-related disorders are responsible for diarrhea-associated TEN (Figure 5). Schneider et al. reported that patients who received *S. cerevisiae* var. *boulardii* can significantly enhance the levels of short-chain fatty acids in 10 TEN patients as compared to the normal controls. This treatment could increase the SCFA level in high stool frequency. The beneficial impact of *S. cerevisiae* var. *boulardii* and the relative decline in TEN-associated diarrhea comparable to control are categorized within the range of 5% and 8.2% in three randomized control trials, respectively [37].

#### 4.1.6. Traveler’s Diarrhea

Traveler’s diarrhea is a common digestive illness that is responsible for frequent stool discharge. It occurs due to the intake of contaminated food or water (Figure 5). Twelve randomized control trials of *S. cerevisiae* var. *boulardii* and other probiotic strains significantly reduced the severity of the infection caused by Traveler’s diarrhea in children (Table 3). The beneficial impact of *S. cerevisiae* var. *boulardii* and the relative decline in Traveler’s diarrhea comparable to the control are categorized within the range of 5% and 11% in two randomized control trials, respectively [58].

### 4.2. Chronic Diseases

#### 4.2.1. Cancer 

*S. cerevisiae* var. *boulardii* is being potentially used to inhibit cancer cell development and progression (Figure 4). It was observed that this probiotic yeast can reduce the tumorigenic effects of colorectal cells in humans. In vivo, high-throughput metagenomic analysis of 281 stool samples confirmed that *S. cerevisiae* var. *boulardii* has significantly inhibited colorectal cancer metastasis by stimulating cancer cell apoptosis and promoting gastrointestinal health via immune modulation. *S. cerevisiae* var. *boulardii* significantly downregulates the expression of various tumor-causing genes including TNFα, Interleukin-1β and Interleukin-17, the expression of NF-_k_B and mTOR signaling cascades was also inhibited (Figure 4). However, the activity of different cytokines was not affected by *S. cerevisiae* var. *boulardii* treatment. HCT116 and DLD1 colorectal cell lines were used to analyze the apoptotic behavior of colorectal cells after the administration of *S. cerevisiae* var. *boulardii*. Results confirmed the presence of an enhanced percentage of apoptosis in probiotic yeast-treated cells [59]. 

#### 4.2.2. Ulcerative Colitis 

Broad-spectrum antibiotics are conventionally used to treat ulcerative colitis but due to antibiotic resistance, their efficacy has been reduced by a substantial level. Probiotics, especially *S. cerevisiae* var. *boulardii* and its derivatives, act as an alternative method for the maintenance of normal gut microbiota and help to treat chronic colitis diseased patients [60]. Studies suggested that the pathogenic strain of *E. coli,* known as adherent-invasive *E. coli* (AIEC), showed a strong binding affinity with the small intestinal lining of Crohn’s disease patients. This Gram-negative bacteria can easily invade the intestine of patients. Patients with Crohn’s disease showed strong adherence between AIEC bacteria due to its FimH adhesion potential and overexpressed mannose residues, which are present on the surface of intestinal glycoprotein CEACAM6 (carcinoembryonic antigen-related cell adhesion molecule). In vivo results reported that *S. cerevisiae* var. *boulardii* significantly blocked the adherence potential of LF82 to the intestinal brush border. Probiotic yeast also lowered the pro-inflammatory cytokine level and was confirmed to treat the pathogenesis of ulcerative colitis [61].

#### 4.2.3. Crohn’s Disease (CD)

Typically, Crohn’s disease is a part of chronic inflammatory bowel disease, which can cause digestive inflammation, abdominal pain, weight loss, watery stool and malnutrition. Individuals with chronic Crohn’s disease may also come across inflammation of skin, liver, joints, anemia, kidney stones and maldevelopment. Bacteria associated with Crohn’s disease can damage the gastrointestinal tract (GI), especially the small intestine, colon and can cause erratic and multiwall GI inflammation. Dalmasso and colleagues reported that consumption of *S. cerevisiae* var. *boulardii* can significantly reduce the level of CD, can control chronic inflammation and reinforce epithelial reformation [62]. In a pilot study, 31 patients with Crohn’s disease were randomly treated with *S. cerevisiae* var. *boulardii* or an antimicrobial drug for 12 weeks. Patients treated with probiotics considerably reduced colonic permeability as compared to the antimicrobial drug-treated patients. Another pilot study of 20 patients with Crohn’s disease, who were administered *S. cerevisiae* var. *boulardii* for 49 days, showed remarkable improvement in the patient’s health (Table 3). *S. cerevisiae* var. *boulardii* consumption after steroidal therapy does not produce health-promoting effects on Crohn’s disease patients [22].

#### 4.2.4. Vaginal Candidiasis

Vaginal Candidiasis can be considered the most common fungus-associated vaginal infection globally. *Candida albicans* is the major causative agent of this disease. Vaginal Candidiasis is typically caused by large antibiotic consumption, which produces fluctuations in the normal composition of the vaginal microbiota. Studies have reported the efficacy of oral and intramuscular administration of *S. cerevisiae* var. *boulardii-*based probiotics [63] (Table 3). Vaginal inoculation of *S. cerevisiae* var. *boulardii* live yeast or inactivated whole yeast can significantly lower the growth of *Candida albicans* in mice vaginas. Both these yeast types cause *S. cerevisiae* var. *boulardii* and fungus interaction which results in prohibiting the cohesion of *Candida albicans* to the vaginal epithelial cells. Probiotic administration can significantly reduce the pathogenicity of *Candida albicans* by lowering its ability to transform itself from yeast to mycelium and the capability of exhibiting aspartyl proteases. However, the efficacy of live yeast is greater as compared to the inactivated whole yeasts [63].

### 4.3. Health Benefits of S. cerevisiae var. boulardii as a Probiotic

#### 4.3.1. Antibacterial and Antiviral Properties

The efficacy of *S. cerevisiae* var. *boulardii* on gastrointestinal microbiota has been critically investigated. *S. cerevisiae* var. *boulardii* can opt for different modes of action for antibacterial and antiviral activities in the human gut, which includes: (i) direct inhibition of pathogenic intestinal microbes and normalizing the pH of the gastrointestinal tract by reducing the pathogenicity of toxic microorganisms, (ii) producing an indirect impact on the gut microenvironment, (iii) producing an immunomodulatory effect on the host body [64]. The antibacterial effects of *S. cerevisiae* var. *boulardii* against different Gram-positive and -negative bacterial and viral pathogens including *Bacillus anthracis*, *Shigella*, *E. coli*, *Vibrio cholera*, *Helicobacter pylori*, *C. difficile*, *Salmonella and* Rotavirus have been previously reported [65]. *S. cerevisiae* var. *boulardii* can adhere to the toxin released by *Vibrio cholera* and inhibit its activity. The enhanced fluidity of sodium and chloride produced by *Vibrio cholera* can significantly be reduced by *S. cerevisiae* var. *boulardii* via inhibition of cyclic adenosine monophosphate-induced chloride secretion. Therefore, probiotic yeast can directly treat *C. difficile* disease by targeting its toxins and receptors. This infection can also be prevented by the action of the protease enzyme of *S. cerevisiae* var. *boulardii* against receptors and bacterial toxins. Moreover, this yeast can also block the pro-inflammatory pathways which are triggered by the toxins of *C. difficile*. It can also inhibit the expression of IL-8 and Erk1/2 genes and the activity of the NF-_K_B pathway (Figure 4) [66]. Anthrax is a bacterial infection that is produced by virulence factors with a protective antigen, lethal factors and edematogenic factors, these peptides are responsible for causing morphological changes in the epithelial cells of the host [67]. The probiotic potential of *S. cerevisiae* var. *boulardii* against *Salmonella enterica Typhimurium* has been analyzed previously. Studies suggested that probiotic yeast can reduce the morbidity and mortality rate of the disease caused by pathogenic *S. Typhimurium* bacteria. It can limit the entry of bacteria into the host intestinal epithelial cells by inactivating the Rac pathway (Figure 4). *S. cerevisiae* var. *boulardii* sticks to the surface of pathogenic bacteria and reduces its multiplication and growth by accelerating bacterial excretion via the stools [68]. *S. cerevisiae* var. *boulardii* can also produce antibacterial properties against peptic ulcer disease caused by Gram-negative *Helicobacter pylori* bacteria, which cause gastrointestinal tract infection and chronic gastric inflammation in the infected stomach. *S. cerevisiae* var. *boulardii* decreases the cytokine and chemokine levels into the stomach and significantly produces IgA antibodies against the toxic *Helicobacter pylori* [66]. This probiotic yeast is also effective against infection caused by viruses including rotavirus. It can suppress the level of oxidative stress in the host cells that are infected with rotavirus and also reduce the Cl^-^ excretion caused by rotavirus [69].

#### 4.3.2. Immune System Modulation

The probiotic effect of *S. cerevisiae* var. *boulardii* on the human immune system has been thoroughly investigated. The mechanisms that are mediated by the action of *S. cerevisiae* var. *boulardii* yeast are: (i) stimulation in the host immune activity, (ii) production of immunoglobulins, (iii) synthesis of cytokines and chemokines, (iv) assistance in the development of immune cells and (v) stimulate immune priming [70]. In a clinical study, *S. cerevisiae* var. *boulardii* administered to a child suffering from gastroenteritis showed a considerable rise in the IgA levels and a reduction in CRP (C-reactive protein) levels. After a 7-day treatment, a significant increase in the rate of CD8 lymphocytes in the *S. cerevisiae* var. *boulardii*-treated group as compared to the control group (Figure 4) [71]. A combination of yeast and bacterial probiotic is capable of treating child-associated diarrhea. Results showed a significant increase in immune system modulation and CD3+, CD4+ and Th1/Th2 levels. Moreover, the clinical outcomes of the diarrheal disease were remarkably increased in the probiotic-treated group [72]. When the pathogenic bacteria enter into the gastrointestinal tract of the host, *S. cerevisiae* var. *boulardii* releases IgA antibodies which bind to the bacteria and excrete it from the host’s body via feces. *S. cerevisiae* var. *boulardii* consumption frequently increased the release of IgA antibodies when the host is exposed specifically to *C. *difficile** toxin A [73]. A study on germ-free mice reported that yeast raised the level of IgM, cytokines and the total number of liver macrophages and cleared the infection caused by pathogenic bacteria from the host intestine in the treated group [74]. Briefly, *S. cerevisiae* var. *boulardii* can mediate different hormonal and molecular responses which are responsible to inhibit the activity of intestinal pathogens. Generally, the defensive mechanism of probiotic yeasts against several toxins is executed by stimulating the production of cytokines and interleukin (IL)-1β, IL-12, IL-6, TNFα, and IL-10 [75]. The in vitro and in vivo studies of *S. cerevisiae* var. *boulardii* showed significant modulation in the host early immune response, through this, the host body can show resistance against most microbial communities. It can also keep the equilibrium between pro and anti-inflammatory immune responses by the upregulation of several cytokines and inhibit the immune cell proliferation and maturation [8]. 

#### 4.3.3. Antioxidant Properties

*S. cerevisiae* var. *boulardii* showed comprehensive antioxidant properties in the previous studies. *S. cerevisiae* var. *boulardii* extracted from the fermentation of guajillo pepper showed 66.1% alleviation in cholesterol level when placed in the incubator for 2 days. In a study, DPPH (1,1-diphenyl-2-picryl-hydrazyl free radical) assay calculated 63% of the total antioxidant potential of *S. cerevisiae* var. *boulardii* [34]. A DPPH scavenging assay of *S. cerevisiae* var. *boulardii* yeast also showed 2.3 mgTE/L antioxidant activity, which is beneficial for the manufacturing of beer with enhanced probiotic potential. Moreover, this assay also exhibited a 40% antioxidant level of *S. cerevisiae* var. *boulardii* yeast extracted from different Brazilian local fermented foods [76,77]. Studies suggested that *S. cerevisiae* var. *boulardii* whole cells possess superior antioxidant properties as compared to its extracts, it can be due to the presence of a high level of 1/3-b-D-glucan in the *S. cerevisiae* var. *boulardii* cell wall structure. Insoluble glucan and metabolites including phenyl ethyl alcohol, vitamin B6, cinnamic acid, vanillic acid and erythromycin are responsible for high antioxidant properties [78]. In a comparative study, raw and miscellaneous *S. cerevisiae* var. *boulardii* extracts were investigated for antioxidant level by DPPH test, superoxide radical scavenging assay by the total number of bioactive compounds. Results of this study confirmed the maximum antioxidant potential of *S. cerevisiae* var. *boulardii* raw extracts as compared to other extracts. *S. cerevisiae* var. *boulardii* antioxidant properties also showed beneficial effects on clinical therapeutics [79]. Another study demonstrated that *S. cerevisiae* var. *boulardii* can induce antioxidant activities of gastrointestinal-induced oxidative stress. In a human organ culture study, *S. cerevisiae* var. *boulardii* showed a reduction in the level of oxidative stress specifically in the rotavirus infected cells via human gastrointestinal examination [80].

#### 4.3.4. Control of Antibiotic Resistance 

*S. cerevisiae* var. *boulardii* showed resistance against both broad and narrow-spectrum antibiotic drugs. However, it cannot resist antifungal drugs and therapies. It is the most suitable and active therapeutic agent for the prevention and medication of all diarrheal-associated diseases which are specifically caused by the fluctuation in the normal gastrointestinal microbiota in patients with continuous antibiotics consumption for a longer run. Furthermore, bacterial probiotics are unable to exhibit such properties [34]. In order to increase the quality of functional food, different probiotic strains have been added to human foods and dietary supplements. The safety of human administered probiotics is significant due to their resistance against various antimicrobials [43]. Antibiotics are considered the fundamental tool to fight against pathogenic bacteria. These pathogenic microorganisms can acquire antibiotic resistance genes against advanced antibiotic drugs [81]. This resistance mechanism can negatively affect the treatment strategy against common bacterial infections [82]. Studies suggested that probiotic bacteria and yeast can act as the reservoir of antibiotic resistance genes. Antibiotic resistance in probiotic strains due to intrinsic or extrinsic mutations does not harm the host gastrointestinal tract. Moreover, they are useful to regain the lost gut microbiota of the host after continuous antibiotic intake. However, these probiotics can horizontally transpose resistance genes in harmful microbes. Tetracyclin and vancomycin resistance genes have been observed in various food and gut microbes [83].

## 5. Mechanisms of Action of *S. cerevisiae* var. *boulardii* Yeast

The responsibility of the host gut microbiota is not limited to just providing protection against pathogenic microbes [84]. It can also contribute to various mechanisms including cellular adhesion, reestablishment of lost gut microbiota, mediation of cancer signaling cascades, competition with pathogenic microbes, mucin production and regulation of nutritional and trophic effects. Adequate administration of *S. cerevisiae* var. *boulardii* can target and eliminate disease-causing microbes from the gastrointestinal tract of the host [85]. 

### 5.1. General Mode of Actions 

Host gut dysbiosis due to the pathogenic microbial attack may reduce the overall probiotic bacterial load in the host gastrointestinal tract which may cause inflammation and secondary infections [86]. The adhesion potential of *S. cerevisiae* var. *boulardii* against pathogenic microbes may actively contribute to neutralizing the mechanism of antigen translocation from the gastrointestinal tract to other parts of the host body [87]. Continuous administration of *S. cerevisiae* var. *boulardii* for several weeks can stabilize the host gut microenvironment by reducing the severity of the disease and eventually eradicating the disease from the host body. Some probiotics are frequently eliminated from the host body, but before the elimination, they would have significantly modulated the host immune system. While other probiotics may recognize and bind to the active sites of the host intestinal mucosal layers [88]. Mucin production by intestinal epithelial cells of the host may also be influenced by the presence of probiotics in the host gut. Both pathogenic and beneficial microbes compete for binding to the gastrointestinal tract of the host. Cell wall proteins and mannose residues of *S. cerevisiae* var. *boulardii* are responsible for the direct binding of probiotic yeast to the intestinal receptors and reducing the probability of pathogenic microbes binding to the active sites [89]. However, if pathogens already adhere to the active sites, then probiotic administration may significantly induce the expression of exogenous sugars which can obstruct the binding of pathogenic microbes to the intestinal mucosal layers.

### 5.2. Mechanisms of Cancer Signaling Cascades

Cancer is considered as a major public health concern globally [90]. It is the leading cause of death not only in developing countries but also in developed countries. This deadly disease is a combination of more than 100 different diseases [91]. There are different types of cancer; all types have the same origin which is the abnormal growth of the cells. The growth rate of healthy and cancerous cells are different, healthy cells grow and proliferate in a controlled manner resulting to keep the body alive, while tumor cells grow in an abnormal fashion leading to cause anti-apoptotic effects [92]. To reduce the expression of oncogenes and protooncogenes, several clinical therapeutics including anticancer drugs, chemotherapy, radiotherapy and other strategies are conventionally used, however, the use of probiotics acts as an alternative treatment method for cancer prevention.

*S. cerevisiae* var. *boulardii* can significantly induce cancer signaling cascades by upregulating the expression of apoptotic proteins and downregulating the expression of protooncogenes and oncogenes. A recent study investigated the anti-tumorigenic activity of *S. cerevisiae* var. *boulardii* against gastric cancer cell lines and analyzed total cellular viability, apoptotic effects and activity of survivin gene after 3 days. Results of this study reported that targeted probiotic yeast significantly reduced the level of cellular viability, which stimulate apoptosis and lowered the activity of the survivin gene in gastric cancer cells (Figure 4). This study strongly recommends the use of *S. cerevisiae* var. *boulardii* as a potential anti-gastric-cancer treatment therapy [93]. The probiotic potential of this yeast was also reported against human colorectal cancer cell lines (HT-29) and animal models. To evaluate the efficacy of *S. cerevisiae* var. *boulardii* on cell growth, development and apoptosis, this yeast was thoroughly spread over the HT-29 cells by using 4′,6-diamidino-2-phenylindole (DAPI) dye and 3-(4,5-dimethylthiazoyl-2-yl)-2,5-diphenyltetrazolium bromide (MTT) assay. The expression profiles of PTEN/caspase-3, Bclxl and RelA genes were evaluated by real-time PCR [94]. After 24 h, the activity of PTEN and caspase-3 gene was increased. However, the expression of Bclxl and RelA genes was significantly reduced (Figure 4). After 2 days, the MTT assay showed inhibition in the growth of probiotic treated HT-29 cells. In another study, 1,3-beta-glucan part of the *S. cerevisiae* var. *boulardii* yeast showed anti-neoplastic effects on rat colon cancer cells when treated with dimethylhydrazine and *S. cerevisiae* var. *boulardii* orally [94]. *Chen* et al. reported that *S. cerevisiae* var. *boulardii* consumption can significantly block the activity of epidermal growth factor receptors when exposed to the targeted yeast and inhibit the Erk and Akt pathway (Figure 4). According to the results, *S. cerevisiae* var. *boulardii* reduced the growth and proliferation of cancer cells and induces cancer cell apoptosis [19].

## 6. Discussion

Probiotics are non-digestible constituents of food, and when added in food or diet, confer useful and healthy effects to the host and stimulate the growth of a confined quantity of colon bacteria [95]. Natural strains of *S. cerevisiae* var. *boulardii* observed harsh environmental conditions as compared to the strains artificially cultured in the lab. This probiotic yeast has advanced conventional survival strategies which ensure its viability for the long run [96]. Mostly, the natural strains of this beneficial yeast are present in the nutrient-enriched soil environment. Some other environmental habitats of *S. cerevisiae* var. *boulardii* are the leaves and trunk surfaces of different medicinal and non-medicinal plants. It is also naturally present in intact grapes and other citrus fruits [27]. The natural transmission of this yeast to the human body is possible by the consumption of grapes, grape wine and different fruits. Studies suggested that this yeast is also insect-borne and is observed in wasps, Drosophila and other insects. These insects absorbed *S. cerevisiae* var. *boulardii* by feeding on the grapes and other fruits [97]. *S. cerevisiae* var. *boulardii* has shown direct and indirect effects on functional (fermented) food stuff. Direct effect indicates host-organism relationship, while indirect effects demonstrate the biogenic upshot (due to taking of microbial metabolites as a result of fermentation). This advances towards the efficient consequences of probiotics that seem to be applied in non-dairy food items as products related to chocolate, chewing gum, biscuit, honey, cereals, cakes, dressing, sweetness and tea [98]. In general, *S. cerevisiae* var. *boulardii* in the food industry somehow has difficulty in its multiplication and survival rate because of the distress conditions of the gastrointestinal tract [99]. 

To ensure the shelf-life of probiotics, novel probiotics are being designed through microencapsulation technology that opposes environmental conditions. Various factors can contribute to the beneficial aspects of probiotics but its proper mechanism of action is still vague. Studies suggested that lactation performance of the dairy animals was improved by *S. cerevisiae* var. *boulardii* yeast supplementation. It is found that the increased milk yield might be due to the stimulatory effect of probiotic yeast on the animal microbiota, which in turn increases cellulose digestion [100]. 

The presence of functional food in animal diets is responsible for increasing the productivity of livestock. Livestock can significantly improve human nutrition by providing essential nutrients in the form of milk, meat, and eggs [101]. An inadequate diet can drastically damage the health of livestock and reduced the overall yield. Due to poor feeding, animals are generally suffering from digestive and respiratory diseases leading to insufficient digestion and consequently retarded growth and productive performance. Dietary supplementation of *S. cerevisiae* var. *boulardii* is a viable and safe option for farmers to enhance the production of lactating dairy cattle and heifers [102]. This yeast has gained the Generally Recognized As Safe (GRAS) status from the Food and FDA, thus, can significantly be used to improve the animal feed supplements [103]. Moreover, the probiotic dose administered to animals is dependent on the (i) composition of feed, (ii) age of the animal, (iii) physical health of the animal and (iv) nature of the digestive system of the animal [29]. 

*S. cerevisiae* var. *boulardii* showed its applications in the wine industry for the benefit of humans. The natural grape was considered as a potential habitat of *S. cerevisiae* var. *boulardii* due to its high sugar content and acidic pH [104]. This yeast can significantly cope with all the fermentation stresses of the environment and has gained “the wine yeast” status and it is considered an important component of the wine industry globally. Moreover, the biosynthesis of primary and secondary alcohols is responsible due to their fermenting ability [105]. *Theobroma cacao* grains are significantly used for the manufacturing of chocolate. Fermentation of cacao grains can reduce the bitter and acrid effects of these grains. Direct exposure of cacao grains to probiotic yeast can induce fermentation reaction resulting in the production of ethanol and useful secondary metabolites [106]. The pectinolytic enzymes of *S. cerevisiae* var. *boulardii* can potentially metabolize citric acid produced by cacao grains. The increased growth of *S. cerevisiae* var. *boulardii* under high pH and stress conditions contributed to ethanol production [104]. *S. cerevisiae* and *S. cerevisiae* var. *boulardii* have remarkable applications in the bread and bakery industry. Sourdough, water and flour mixture are required for the manufacturing of bread. Various types of flours are commercially available which include spelt, barley, maize, einkorn, rye, khorasan, sorghum and many others [107]. Probiotic yeast and lactic acid bacteria are the main components of sourdough. A total of 2% of the fermenting yeast is added for the biosynthesis of bread. Atmospheric oxygen enters into the dough during dough mixing, which is adequately consumed by the yeast cells. Moreover, in an oxygen-limited environment, the rate of yeast cell reproduction was hindered and dough started to rise due to the fermentation process [108].

## 7. Conclusions

Continuous upsurge in multidrug-resistant organisms is responsible for causing millions of deaths annually. To control the spread of antimicrobial resistance, probiotic yeast *S. cerevisiae* var. *boulardii* can be considered as an alternative method for the treatment of bacterial and fungal infections. Several clinical and therapeutic studies confirmed the efficacy of *S. cerevisiae* var. *boulardii* against different pathogenic gastrointestinal diseases. The probiotic nature of this yeast has surpassed the effectiveness of different probiotic bacteria due to its gut microbiota protection potential. This probiotic yeast can actively participate in the manufacturing of bread, bakery products, wine, chocolate and large-scale bioethanol production. The consumption of *S. cerevisiae* var. *boulardii* in adequate amounts can also enhance the overall yield of milk and meat in poultry and livestock. The prescribed *S. cerevisiae* var. *boulardii* dose can also reduce the probability of co-morbidities that are caused by the continuous consumption of antibiotics for a long period. The combination of *S. cerevisiae* var. *boulardii* with other probiotics can enhance the treatment efficacy and reduce the pathogenicity of the disease. Despite its beneficial aspects, the use of this probiotic yeast should be according to the prescription of a physician. Moreover, this study will open up new insights for the development of novel probiotic strains, which will reduce the transmission of antimicrobial resistance genes among humans and farm animals.

## Figures and Tables

**Table 1:** Commercially available probiotic yeast products.

Commercial Probiotic Product	Probiotic Strain	Serving per Pack	Company	Country	Dose per Capsule or Tablet	Stability at 25 °C	Colony Forming Unit (CFU)	Strain Specific Studies	Cost
Reflor (Single strain)	*Saccharomyces boulardii lyo*	10 Capsules	Biocodex	Turkey	250 mg	✓	5 × 10^9^ CFU	✓	16$
Inteflor	*Saccharomyces boulardii* + 1 bacterial probiotic strain	30 Capsules	Yamamato Research	United States	150 mg	✓	15 × 10^9^ CFU	×	20$
Ultimate Flora Pobiotic	*Saccharomyces boulardii* + 10 bacterial probiotic strains	30 Capsules	RenewLife	United States	----	✓	25 × 10^9^ CFU	×	16$
Florstor	*Saccharomyces boulardii lyo*	20 Capsules	Biocodex	United States	250 mg	✓	5 × 10^9^ CFU	✓	20$
Saccharomyces Boulardii	*Saccharomyces boulardii*	100 Capsules	Kirkman	United States	150 mg	×	3 × 10^9^ CFU	×	39$
Saccharomyces Boulardii	*Saccharomyces boulardii*	120 Capsules	Allergy Research Group	United States	150 mg	×	3 × 10^9^ CFU	×	12$
Saccharomyces Boulardii 10B	*Saccharomyces boulardii*	120 Capsules	Pure Therapro Rx	United States	----	×	10 × 10^9^ CFU	×	21$
Nexabiotic	*Saccharomyces Boulardii*	60 Capsules	DrFlormulas	United States	150 mg	✓	17×10^9^ CFU	×	21$
Flora	*Saccharomyces bolourdii*	30 Capsules	Institute Rosell Lafelmanol	Belgium	----	✓	10 × 10^9^ CFU	✓	14$
Daily Probiotic	*Saccharomyces boulardii*	100 Capsules	Florastor	United States	250 mg	✓	10 × 10^9^ CFU	×	19$
Saccharomyces Boulardii PLUS MOS	*Saccharomyces boulardii*	90 Capsules	Jarrow Formulas	United States	----	✓	5 × 10^9^ CFU	×	22$
Perenterol	*Saccharomyces boulardii lyo*	50 Capsules	Biocodex	Germany	250 mg	✓	5 × 10^9^ CFU	✓	20$

**Table 2:** Parameters for the survival of *S. cerevisiae* var. *boulardii*.

Sr. No.	Survival Parameter	Optimum Value	Reference
1	Temperature	22–30 °C	[46,47]
2	Water Activity	0.98%	[48]
3	pH and Acidity	2–8	[29,47,49]
4	Nutrient Media	YEPD, OGA, SDA	[49,50]

**Table 3:** Per capsule/tablet dose of *S. cerevisiae* var. *boulardii* for the treatment of different acute and chronic diseases.

Acute Diseases	Dose per Capsule	Treatment Duration	Reference	Chronic Diseases	Dose per Capsule	Treatment Duration	Reference
Antibiotic-Associated Diarrhea	1000 mg	14 Days	[44]	Cancer	1000 mg + Cancer Specific Drug Doses	30 Days	[50]
*Clostridium difficile* Infection	1000 mg	30 Days	[45]	Ulcerative Colitis	1000 mg	14 Days	[51]
Acute Diarrhea	750 mg	1 Week	[46]	Crohn’s disease	750 mg	49 Days	[22]
Persistent Diarrhea	1000 mg	14 Days	[47]	Vaginal Candidiasis	500 mg	30 Days	[52]
Enteral Nutrition-related diarrhea	2000 mg	30 Days	[48]				
Traveler’s Diarrhea	250 mg	21 Days	[49]				

## Data Availability

Not applicable.
